# Mo:BiVO_4_ Nanoparticles-Based Optical Modulator and Its Application in a 2-μm Pulsed Laser

**DOI:** 10.3390/nano11123243

**Published:** 2021-11-29

**Authors:** Lina Zhao, Wenyu Zhang, Ye Yuan, Luyang Tong, Jingjing Liu, Jie Liu, Yangjian Cai, Yuanmei Gao

**Affiliations:** 1 Center of Light Manipulations and Applications, College of Physics and Electronics, Shandong Normal University, No 88, East Wenhua Road, Jinan 250014, China; lnzhao@sdnu.edu.cn (L.Z.); 2020020563@stu.sdnu.edu.cn (W.Z.); 2019020530@stu.sdnu.edu.cn (Y.Y.); 2020010065@stu.sdnu.edu.cn (L.T.); jingjingliu@sdnu.edu.cn (J.L.); Jieliu@sdnu.edu.cn (J.L.); 2 Shandong Provincial Key Laboratory of Optics and Photonic Device, No 88, East Wenhua Road, Jinan 250014, China; 3 School of Physical Science and Technology, Soochow University, Suzhou 215006, China

**Keywords:** pulsed laser, optical modulator, saturable absorber, nanoparticles

## Abstract

Mo:BiVO_4_ nanoparticles were employed as an optical modulator in a Q-switched all-solid-state Tm:YAP laser for the first time. The nonlinear optical parameters of Mo:BiVO_4_ nanoparticles in the 2-μm region were characterized by measuring nonlinear transmission. Saturation intensity was 718 MW/cm^2^, and the modulation depth was 12.3%. A stable pulse sequence was acquired with a 70.08 kHz maximum repetition rate and an 821 ns pulse width. The maximum output average power was 153 mW, corresponding to 2.18 μJ single pulse energy and 2.67 W peak power. Although the response wavelength of Mo:BiVO_4_ is in visible light region, our experimental results demonstrates that a saturable absorption effect for wavelengths much longer than visible light (2 μm wavelength) is still possible due to sub-bandgap absorption. Therefore, we experimentally proved that Mo:BiVO_4_ nanoparticles are a great candidate for use as an optical modulator of a 2-μm pulsed laser.

## 1. Introduction

Semiconductor-based optical modulators in a pulsed laser field have been developed for a few decades. This type of optical modulators started from semiconductor saturable absorber mirrors (SESAMs), which were fabricated by InGaAs/GaAs or InGaAs/GaSb multiple-quantum-well layers and extensively utilized to generate short pulses in the spectral region from near-infrared (IR) to mid-IR wavelengths [1,2,3,4]. However, the manufacturing procedures for SESAMs are complex. In recent years, semiconductor material-based nanostructures have been extensively investigated and reported on due to their excellent chemical and physical characteristics as well as their ability to overcome the complicated fabrication problems of SESAMs. IV–VI group nanoparticles (NPs), including PbS and SnSe, have been used as optical modulators to generated pulsed lasers [5,6]. Liu et al. employed PbS NPs as the saturable absorber (SA) to modulate a Tm-doped fiber laser and obtained a 2-μm ultrafast laser [5]. Ma et al. reported on a passively mode-locked Tm-doped fiber laser using SnSe NPs as SA [6]. III–V group boron nitride (BN) nanosheets were employed to generate a passively mode-locked Tm: YAP laser [7]. Members of the II–VI group, such as ZnO, CdS, and especially ZnO NPs, have a short recovery time of 1–5 ps and a high third-order nonlinear coefficient, and therefore represent ideal SAs for pulsed lasers. Ahmad et al. reported a Q-switched Er-doped fiber laser for 1.55 μm operation [8] and a mode-locked Tm-doped fiber laser using ZnO NPs for 2 μm operation [9]. CdS quantum dots as SA have been used in an Er-doped fiber laser to generate Q-switched pulses [10]. Besides ZnO, the use of oxide semiconductors such as TiO_2_, Al_2_O_3_, NiO, and Fe_3_O_4_ NPs as optical modulators has also been demonstrated in previous reports [11,12,13,14,15].

In addition, due to their excellent chemical and physical characteristics, other types of semiconductors could potentially be applied in pulsed lasers. In the last decade, as a visible-light-driven semiconductor, BiVO_4_ has become the most promising material, since it has been widely applied in several fields, including the reduction of CO_2_ [16,17,18], the degradation of organic compounds [19,20], and O_2_ generation from water splitting [21,22,23]. Therefore, it is recognized as an effective photocatalyst. BiVO_4_ possesses the advantages of non-toxicity, low production cost, and great stability. The moderate band gap is 2.4 eV, corresponding to the response wavelength in the visible light region [24,25]. The charge relaxation time was reported to be as short as 40 ps by using broadband transient absorption spectroscopy [26]. However, BiVO_4_ suffers from several challenging issues, such as a low separation rate and mobility of photogenerated electron-hole pairs [27,28]. By doping with electron-rich elements such as Mo^6+^ or W^6+^, the performance is greatly improved, owing to the increased electron density [29,30]. The bandgap of Mo:BiVO_4_ is approximately equal to that of BiVO_4_ [31]. All of these characteristics indicate that Mo:BiVO_4_ fulfills the requirements of an optical modulator. Although the response wavelength of Mo:BiVO_4_ is in the visible light region, the saturable absorption effect for wavelengths that are longer than visible light is possible due to sub-bandgap absorption, which could be attributed to boundary effects, edges, and defects. The sub-bandgap absorption effect has been proved in other materials, such as ZnO particles and few-layer MoS_2_ [32,33]. Our group also demonstrated that Mo:BiVO_4_ nanoparticles could perform as an optical modulator in a 1-μm pulsed laser, which is the first demonstration of the application of Mo:BiVO_4_ nanoparticles in a pulsed laser [34].

Recently, 2-μm pulsed lasers have attracted much attention due to their excellent performance in the fields of laser medicine, imaging, optical communication, and sensing [35,36,37]. Numerous semiconductor nanoparticles have been utilized as saturable absorbers for 2-μm pulsed lasers, such as SnSe [6], BN [7], ZnO [9], Fe_3_O_4_ [14], and so on. As far as we know, Mo:BiVO_4_ nanoparticles have not been applied in 2-μm pulsed lasers in previous works.

In this study, Mo:BiVO_4_ NPs were employed as a SA in a Q-switched all-solid-state Tm:YAP laser for the first time. The nonlinear optical parameters of Mo:BiVO_4_ NPs in the 2-μm region were characterized by measuring nonlinear transmission. Stable Q-switched lasers at 1937.9 nm were realized with a 70.08-kHz maximum repetition rate and 821-ns pulse width. The delivered maximum average power was 153 mW, corresponding to 2.18 μJ pulse energy and 2.67 W peak power.

## 2. Materials and Methods

A Mo:BiVO_4_ thin film is fabricated by a sol-gel and calcining method [38,39]. The synthesis process of Mo:BiVO_4_ was as follows: 0.2 mol Bi(NO_3_)_3_-5H_2_O was dissolved in glacial acetic acid, thereby forming solution 1. Solution 2 was obtained by dissolving a certain quality of vanadium oxy acetylacetonate. Solution 3 was formed by dissolving 0.2 mol acetylacetone molybdenum in acetylacetone solution. After stirring, the three solutions were mixed together on the basis of the metal molar proportion Mo:V:Bi = 0.01:0.99:1. The mixed solution was set under supersonic condition and taken out after 40 min. After 6 h, the Mo-doped BiVO_4_ precursor liquid with Mo content of 1% (molar wt) was obtained. Next, 380 μL of precursor liquid was taken out and spin-coated on a quartz sheet with a rotating speed of 700 rpm for 10 s. The thin film on the quartz sheet was put in the oven at a temperature of 150 °C for 10 min, calcined in the muffle furnace at a temperature of 470 °C for 30 min, and the above procedure was repeated three times. Mo:BiVO_4_ NPs SA was obtained.

**Figure 1:**
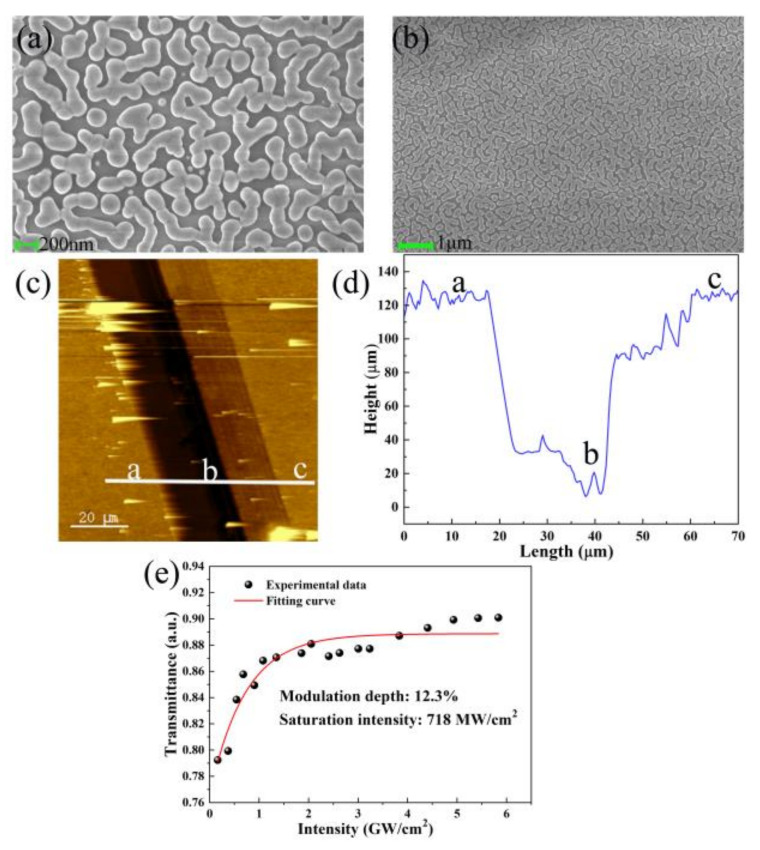
Characterizations of Mo:BiVO_4_ nanoparticles dispersion and nonlinear transmission: (**a**) the SEM with a 200 nm scale; (**b**) the SEM with a 1 μm scale; (**c**) the atomic force microscopy (AFM) image, where position b is the area with no film, and positions a and c are the areas with film; (**d**) the height of the Mo:BiVO_4_ nanoparticles; (**e**) the nonlinear transmission.

The morphology of Mo:BiVO_4_ NPs was measured using a field emission scanning electron microscope (FE-SEM) (ZEISS, sigma500, Oberkochen, Baden-Württemberg, Germany). Figure 1a,b shows the morphology of the particles in different scales, indicating that they are uniformly distributed. Porous structures consisting of uniform particles were observed on the surface of the Mo:BiVO_4_ films. Atomic force microscopy (AFM) (SmartSPM, HORIBA, Kyoto, Japan) was employed to measure the thickness of the Mo:BiVO_4_ NPs sample, as shown in Figure 1c,d. To measure the thickness of the sample, a knife was used to remove the film at position b. The thickness difference between position a, b, and c is shown in Figure 1d. At position b with no film, the thickness was close to zero, and at position a and c with film, the thickness was about 125 nm. The same heights at position a and c indicates that the film was uniformly distributed. A femtosecond pulsed laser at 1935 nm with a 5-MHz repetition rate and a 500-fs pulse width was utilized to characterize the nonlinear transmission of the SA of the Mo:BiVO_4_ NPs. By gradually increasing the incident laser power, the corresponding transmission versus incident laser intensity was recorded, as shown in Figure 1e. The saturable absorption formula was used to fit the experimental data.

(1) $$T=1-\Delta D*\exp(-\frac{I}{I_{s}})-\alpha_{ns}$$

Here, *T* is transmission, Δ*D* is the modulation depth, *I* and *I_s_* are incident fluence and saturation intensity, respectively, and *α*_ns_ is non-saturable loss. The fitting results show that the saturation intensity is 718 MW/cm^2^ and the modulation depth is 12.3%.

## 3. Results

**Figure 2:**
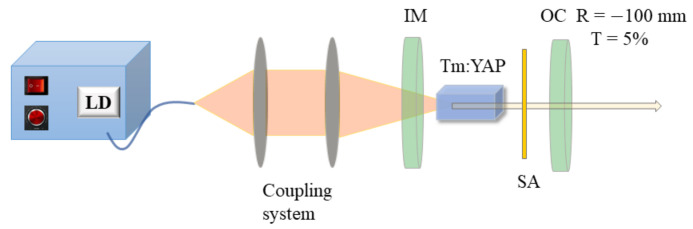
The schematic of the 2-μm pulsed laser experimental setup. LD is laser diode, IM is the input mirror, Tm:YAP is the laser crystal, SA is saturable absorber, OC is the output coupling concave mirror with radius curvature of 100 mm (R = −100 mm) and transmissivity of 5% (T = 5%).

Figure 2 shows the schematic of the 2-μm pulsed laser experimental setup. A fiber-coupled continuous wave (CW) laser diode (LD) with 793 nm central wavelength was employed as the pump light (core size: 105 μm, numerical aperture: 0.22). The cavity was composed of an input mirror and output coupling mirror. A plane mirror was utilized as the input mirror (IM), which was anti-reflectively (AR) coated at 790–800 nm and high-reflectively (HR) coated at 1.9–2.1 μm. The output coupling mirror (OC) was a concave mirror whose radius of curvature was 100 mm and partial-reflectively coated (transmissivity: T = 5%) at 1.9–2.1 μm. The laser crystal was an uncoated 3 at.% Tm-doped YAP (dimensions: 3 mm × 3 mm × 10 mm). The beam from the end of the fiber was imaged onto the laser crystal through a 1:2 coupling system. The Mo:BiVO_4_ SA was inserted between the Tm:YAP crystal and OC.

**Figure 3:**
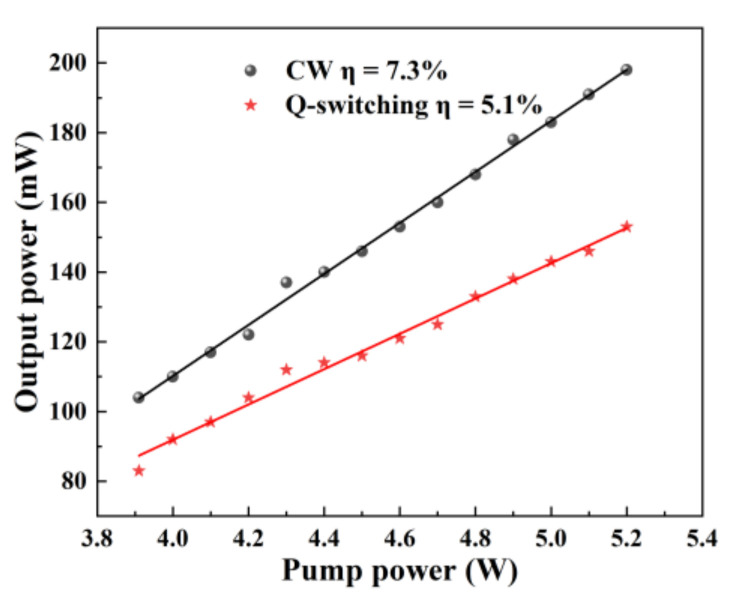
The average output power of the CW and pulsed Tm:YAP laser.

First, the output power of the CW Tm:YAP laser was maximized by adjusting the cavity under 3.91 W pump power. The cavity was then fixed to survey the influence of pump power on the output power. The output power reached 198 mW with a slope efficiency of 7.3% under 5.2 W launched pump power, as shown in Figure 3. Mo:BiVO_4_ SA was then inserted into the cavity. A stable Q-switched laser (QSL) was achieved. Due to the insertion of SA, the loss of the cavity in QSL was higher; thus, the output power of Tm:YAP laser in QSL operation was lower than that in CW operation. Under QSL operation, 153 mW average power was acquired with a slope efficiency of 5.1%.

**Figure 4:**
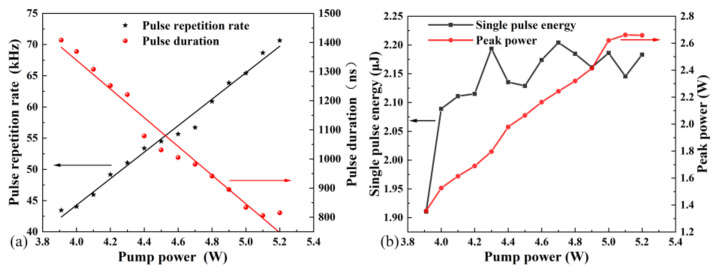
(**a**) Pulse repetition rate frequency and pulse duration of the PQSL. (**b**) Single pulse energy and peak power of the PQSL.

A detector (Electro-Optics Technology, ET-5000, Traverse City, MI, USA) with 28 ps rise time and an oscilloscope (LeCroy, HDO4104A, New York, NY, USA) with 1 GHz bandwidth were employed to measure the pulse sequence of the pulsed Tm:YAP laser. The pulsed sequence well manifested the characteristics of pulsed laser. With an increase in pump power, the repetition rate frequency tended to be larger and the pulse duration tended to be narrower. When the power of the pump source was increased from 3.9 to 5.2 W, the repetition rate frequency increased from 43.44 to 70.08 kHz, while the pulse width reduced from 1.4 μs to 821 ns, as shown in Figure 4a. The single pulse energy and peak power were calculated according to the output power, pulse width, and repetition rate. The dependence of peak power and single pulse energy on the incident power are shown in Figure 4b. The peak power and single pulse energy increased with the increase in pump power. When the pump power was higher than 5 W, the single pulse energy tended to be saturated, which was mainly due to the thermal effect on the SA. The single pulse energy was calculated to be 2.18 μJ, and peak power was 2.67 W under 153 mW average power.

**Figure 5:**
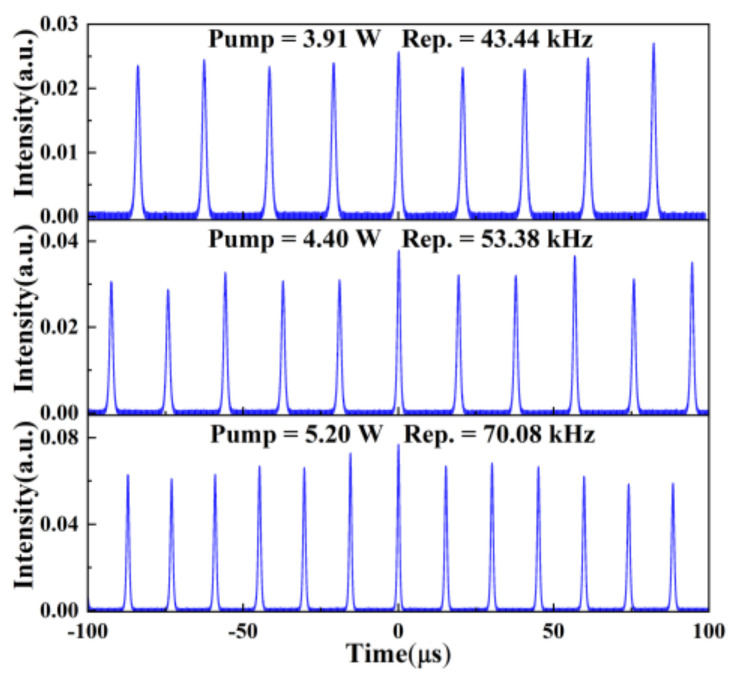
Temporal pulse sequences at different pump powers.

**Figure 6:**
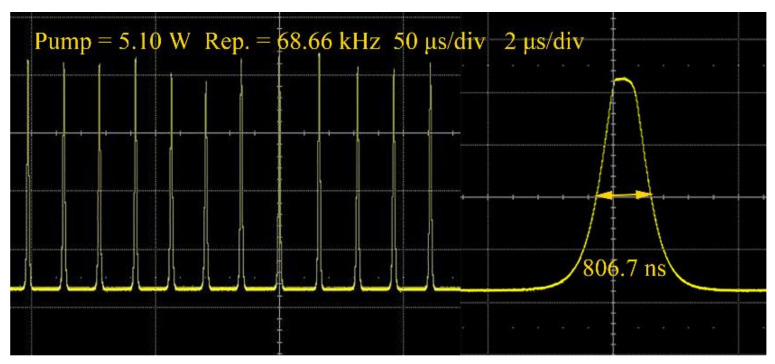
The shortest pulse at different time scales.

The temporal pulse profiles at different repetition rate frequencies were measured, as shown in Figure 5. The pulse sequences at different pump powers (3.91, 4.4, and 5.2 W) were summarized for comparisons. The pulses durations under pump powers 3.91, 4.4, and 5.2 W were 1.41, 1.08, and 0.821 μs, respectively, corresponding to the repetition rate frequencies of 43.44, 53.38, and 70.08 kHz, respectively. The minimum pulse duration was 806.7 ns. The pulse sequences at different time scales are shown in Figure 6.

**Figure 7:**
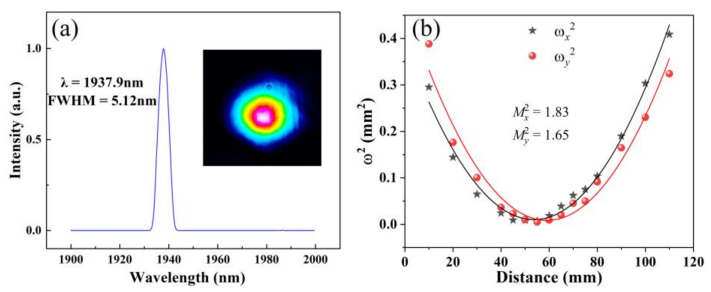
(**a**) The spectrum of the 2-μm pulsed laser (the insert picture is the beam profile). (**b**) Beam quality M^2^ factors.

The central wavelength of the Q-switched laser was recorded by an optical spectral analyzer. Figure 7a shows that the central wavelength was 1937.9 nm with the full width at half maximum of 5.12 nm. The insert picture was the beam profile of the pulsed laser recorded by a CCD camera, which had a Gaussian-like profiles. A focus lens with focal length of 200 mm was employed to measure the beam quality of the output pulsed laser. The beam radii were measured before and behind the focus point, and were fitted by using a parabolic curve, as shown in Figure 7b. After fitting, the *M*^2^ factors were $M_{x}^{2}=1.83$ and $M_{y}^{2}=1.65$ (Figure 7b).

## 4. Discussion and Conclusions

In conclusion, Mo:BiVO_4_ NPs were employed as a SA in a Q-switched all-solid-state Tm:YAP laser for the first time. The nonlinear optical parameters of Mo:BiVO_4_ NPs in the 2-μm region were characterized by measuring nonlinear transmission. Saturation intensity was 718 MW/cm^2^, and the modulation depth was 12.3%. A stable pulse sequence was acquired with 70.08-kHz maximum repetition rate and 821-ns pulse width. The maximum output average power was 153 mW, corresponding to 2.18 μJ single pulse energy and 2.67 W peak power. Although the response wavelength of Mo:BiVO_4_ is in the visible light region, our experimental results, combined with our previous reports [34], further demonstrate that the saturable absorption effect for wavelengths longer than visible light (infrared wavelength) is still possible due to sub-bandgap absorption. Even in the 2-μm region, the photon energy was 0.62 eV, which is far lower than the bandgap Mo:BiVO_4_ NPs (2.4 eV), the Mo:BiVO_4_ NPs still perform well. Therefore, we experimentally proved that Mo:BiVO_4_ NPs are a great candidate for use as an optical modulator for a 2-μm pulsed laser.
